# Methicillin-resistant Staphylococci in canine pyoderma in Thailand

**DOI:** 10.14202/vetworld.2023.2340-2348

**Published:** 2023-11-27

**Authors:** Putu Ayu Sisyawati Putriningsih, Patchara Phuektes, Suphattra Jittimanee, Jaruwan Kampa

**Affiliations:** 1 Graduate School, Faculty of Veterinary Medicine, Khon Kaen University, Khon Kaen, 40002, Thailand; 2 Laboratory of Veterinary Internal Medicine, Faculty of Veterinary Medicine, Udayana University, Bali, 80361, Indonesia; 3 Division of Pathobiology, Faculty of Veterinary Medicine, Khon Kaen University, Khon Kaen, 40002, Thailand

**Keywords:** antibiogram, methicillin-resistant, prevalence, risk factor, Staphylococci, Thailand

## Abstract

**Background and Aims:**

Methicillin-resistant Staphylococci (MRS) seriously threatens animal and human health. Repeated antibiotic use allows the bacteria to develop resistance to several antibiotic classes and become multidrug-resistant (MDR). Canine pyoderma, a common skin condition in dogs, is mainly caused by Staphylococci, including MRS. Detecting this infection in all canine populations is crucial to develop a proper preventive plan. This study estimated the prevalence, antibiogram, and risk factors of MRS in canine patients at a referral animal hospital in Khon Kaen, Thailand.

**Materials and Methods:**

Skin swabs and relevant information were collected from 56 client-owned dogs that visited the hospital from September 2019 to September 2020. Staphylococci colonies were subjected to molecular identification and antibiotic susceptibility tests using an automated system (VITEK^®^ 2). These colonies were also genetically identified using multiplex-polymerase chain reaction (PCR) and sequencing. The *mec*A gene, encoding methicillin resistance, was detected using simplex-PCR. The risk factors of MRS infection and their association with MRS infection were analyzed using logistic regression and the Chi-square test, respectively.

**Results:**

The prevalence of MRS was found to be 35.7% (20/56 dogs). By species, methicillin-resistant *Staphylococcus pseudintermedius* was found in 24 of 104 isolates (23.1%), and all samples were MDR. Receiving systemic antibiotics in the past 6 months was a major risk factor associated with MRS infection (p < 0.05; odds ratio (OR) > 1). In addition to the MRS isolates, the *mec*A gene was also detected in methicillin-susceptible Staphylococci isolates. This might be because of the high expression of *bla*I, and mutations in c-di-AMP cyclase DacA, RelA, and Fem proteins.

**Conclusion:**

A high prevalence of MRS and MDR was observed in the studied population, which might be potentially due to improper antibiotic use by the owners and horizontal transfer of drug-resistance genes.

## Introduction

Antimicrobial resistance (AMR) is a serious threat to humans and animals. Certain bacteria can develop resistance to a particular antimicrobial class, such as β-lactam antibiotics. Methicillin-resistant Staphylococci (MRS) survive under antibiotic treatment and exacerbate the infection. Bacteria resistant to two or more antibacterial classes are considered multidrug-resistant (MDR), which further limits the drug selection for effective treatment [1]. Pets are well-known AMR reservoirs that transfer mobile genetic elements, which confer AMR in other species, including humans [2, 3]. According to Song *et al*. [4], the proximity between animals and their owners provides mixed hosts for these microorganisms. Consequently, antibiotic misuse in veterinary medicine can directly impact humans [5, 6].

Methicillin-resistant Staphylococci are considered as important pathogens worldwide [7, 8]. They can persist in animals and various environments [9] for prolonged periods, and potentially infect humans [10]. Genomic analysis revealed that Staphylococci species originating from different hosts and environments can mutually exchange several resistance factors [11]. Methicillin-resistant Staphylococci carry the *mec*A gene that encodes a modified penicillin-binding protein 2a, which confers resistance to β-lactam antibiotics [12].

Canine pyoderma is mainly caused by *Staphylococcus pseudintermedius* [13], a member of coagulase-positive Staphylococci (CoPS). This group includes *Staphylococcus aureus* and *Staphylococcus schleiferi* found in cutaneous infections in dogs [7]. Among the coagulase-negative Staphylococci (CoNS) groups, which are less pathogenic than CoPS, *Staphylococcus haemolyticus*, *Staphylococcus epidermidis*, and *Staphylococcus saprophyticus* have been isolated from various clinical samples from dogs and cats [14]. However, CoPS and CoNS possess several AMR genes found in mobile genetic elements, which confer resistance [15]. In general, Staphylococcal infections on canine skin can be effectively treated using topical therapeutics. Combined with topical treatment, systemic antibiotics are suggested for superficial pyoderma with a large lesion distribution. The guidelines for diagnosis and antimicrobial therapy of canine superficial bacterial folliculitis recommend a list of systemic antibiotics for empirical use, classified as tier 1. Tier 2 is a list of antibiotics based on susceptibility tests that can be used when no tier 1 options are available [16]. However, several underlying causes, such as allergies, hormones, ectoparasites, and use of immune suppressive drugs, lead to repeated antibiotic use, resulting in MRS or MDR [17–20]. Therefore, canine pyoderma treatment should target the infections and underlying causes of the recurrent problem. The data on MRS infections are rapidly increasing worldwide, including in Thailand. Although methicillin-resistant *S. pseudintermedius* (MRSP) and MDR have been reported in canine patients in Bangkok and other municipal cities [21–23] in central Thailand, clinical information from other areas is limited.

This study aimed to estimate the prevalence of MRS and MDR infections in dogs with pyoderma, investigate the pattern of antibiotic susceptibility and the antibiogram, and identify the risk factors in Khon Kaen province in northeastern Thailand.

## Materials and Methods

### Ethical approval

This study was approved by the Institutional Animal Care and Use Committee of Khon Kaen University with the number IACUC-KKU-39/62.

### Study period and location

The study was conducted from September 2019 to September 2020 with 56 owned dogs with pyoderma who visited the Veterinary Teaching Hospital, Khon Kaen University (VTH-KKU), the referral center for animal dermatology of the region.

### Study population

The dog’s information, including sex, breed, age, reproductive status, allergies, and having steroids, was gathered along with the previous antibiotic use within 2 years before the sample collection date. With pyoderma lesions, two skin swabs were collected from various dog sites and placed in a transport medium, and stored at 4°C before culture primarily on 5% sheep blood agar.

### Bacterial identification and antibiotic susceptibility testing

A primary culture of each skin swab sample was grown on sheep blood agar at 37°C for 18 h–48 h. The colonies resembling *Staphylococcus* were then subcultured and tested using Gram’s staining, slide catalase test, and oxidase test. From each plate, a single colony containing Gram +, catalase-positive, and oxidase-negative cocci was subcultured on a fresh sheep blood agar plate. The isolated colonies were subsequently stored for phenotypic identification and antibiotic susceptibility test (AST) using the VITEK^®^ 2 system (bioMérieux, Marcy l’Etoile, France). The Gram-positive card of the VITEK^®^ 2 system contains 43 biochemical tests to determine the utilization of carbon sources, enzyme activities, and resistance. The reports on bacterial identification had six confidence levels: Excellent, very good, good, acceptable, low discrimination, and inconclusive or unidentified microorganisms [24]. Antibiotic susceptibility test was performed using the AST card based on the microdilution method to determine the antibiotic’s minimum inhibitory concentration (MIC) value against the bacteria. The tested antibiotics included oxacillin, benzylpenicillin, amoxicillin/clavulanic acid, cephalothin, cefpodoxime, cefovecin, amikacin, gentamicin, enrofloxacin, marbofloxacin, pradofloxacin, erythromycin, clindamycin, doxycycline, minocycline, nitrofurantoin, chloramphenicol, florfenicol, and trimethoprim/sulfamethoxazole (SXT). Methicillin-resistant Staphylococci and inducible clindamycin Staphylococci were identified based on an MIC ≥ 0.5 μg/mL for oxacillin, which indicates methicillin resistance. Bacterial growth in the wells of the microtiter plate indicated resistance to Lincosamides (clindamycin, lincomycin, and pirlimycin), as suggested by the Clinical and Laboratory Standards Institute [25].

### Genotypic identification of Staphylococci

DNA was extracted from the isolated Staphylococci colonies using a GF-1 Bacterial DNA extraction kit (Vivantis, Malaysia). Sasaki *et al*. [26] developed a multiplex-PCR for identifying CoPS based on the amplification of the *nuc* gene locus [27]. Other molecular methods including the simplex-PCR method by Shome *et al*. [28] or 16s ribosomal RNA (rRNA) gene sequencing method by Greisen *et al*. [29] were used for molecular identification to identify any CoNS due to false positive slide coagulase results from the shared capsular antigens [27].

### Identification of MRS using *mecA*-PCR

The *mecA* gene was identified in the Staphylococci species using conventional PCR based on the protocol of Oliveira and de Lencastre [30]. The forward and reverse primers were: MECA P4; 5’-TCCAGATTACAACTTCACCAGG-3’ and MECA P7; 5’-CCACTTCATATCTTGTAACG-3’, respectively. For analysis, the DNA fragments were electrophoresed on a 1.5% agarose gel in 1× Tris-acetate-ethylenediaminetetraacetic acid buffer and stained with Redsafe™ (iNtRON Biotechnology, Korea).

### Statistical analysis

The prevalence of MRS infection was calculated using the formula by Noordzij *et al*. [31]. The antibiogram of MRS and methicillin-susceptible Staphylococci (MSS) are presented descriptively. The level of agreement between the results from VITEK^®^ 2 and the molecular techniques used to identify the *Staphylococcus* spp. was checked using Kappa analysis. The efficacy of the automated method was evaluated using predictive values, positive and negative, corresponding to the actual prevalence. The risk factors of MRS infection were investigated using the logistic regression method (IBM SPSS Statistics, Version 25.0, IBM Corp., NY, USA), and their association with MRS infections, assessed using VITEK^®^, was analyzed using the Chi-square test.

## Results

### Study population

Samples comprised 112 swabs collected from 29 female and 27 male dogs. The median age of the dogs was 6-years-old, with the following age distribution: < 2 years (4 dogs), 2–6 years (27 dogs), 7–11 years (18 dogs), and > 11 years (7 dogs). Of these, 40 dogs belonged to 16 different pure breeds, while 13 were mongrels. The records showed that 2 years before the sampling date, 11 dogs never had pyoderma, 15 had pyoderma once or twice, and 30 had more than 3 times. Further, 28 dogs were diagnosed with allergies and 18 dogs received immune-modulating/immune-suppressive medicine for pruritic control or other health problems, such as tumors.

### Identification of Gram-positive cocci

Of the 112 collected skin swab samples, 104 samples had Staphylococcal-like colonies. Using the VITEK^®^ 2 system, five Staphylococci species were identified among these 104 samples: 81 with *S. pseudintermedius* (78%), 13 with *S. schleiferi* (12%), 5 with *S. haemolyticus* (5%), 4 with *S. aureus* (4%), and 1 with *Staphylococcus warneri* (1%). Among the 104 isolates, six Staphylococci and one Macrococci species were identified using molecular methods. Multiplex-PCR revealed that 82 isolates belonged to *S. pseudintermedius* (79%), 12 to *S. schleiferi* (11%), and 3 to *S. aureus* (3%). Five of seven negative multiplex-PCR isolates were identified as *S. haemolyticus* (5%) using the simplex-PCR method for CoNS. One isolate each of *Staphylococcus condimenti* and *Macrococcus canis* was identified by sequencing the amplified 16s rRNA gene. Table-1 compares the species identified using phenotypic and molecular methods.

**Table-1:** Comparison identification results between the VITEK^®^ 2 system and molecular methods of Staphylococcal-like colonies isolated from 56 canine pyoderma cases from September 2019 to September 2020 at Veterinary Teaching Hospital, Khon Kaen University.

VITEK system	Molecular identification							

	*S. pseudintermedius*	*S. schleiferi*	*S. aureus*	*S. haemolyticus*	*S. warneri*	*S. condimenti*	*Macrococcus canis*	Total
*S. pseudintermedius*	81	0	0	0	0	0	0	81
*S. schleiferi*	0	12	0	0	0	1	0	13
*S. aureus*	1	0	3	0	0	0	0	4
*S. haemolyticus*	0	0	0	5	0	0	0	5
*S. warneri*	0	0	0	0	0	0	1	1
Total	82	12	3	5	0	1	1	104

*S. pseudintermedius=Staphylococcus pseudintermedius, S. schleiferi=Staphylococcus schleiferi, S. aureus=Staphylococcus aureus, S. haemolyticus=Staphylococcus haemolyticus, S. warneri=Staphylococcus warneri*

The level of agreement between the VITEK^®^ 2 system and molecular techniques was assessed using Kappa analysis. Overall, the VITEK^®^ 2 system showed a Kappa value of 0.921, which is in almost perfect agreement with the molecular methods. The results slightly disagreed for one isolate each of *S. aureus*, *S. schleiferi*, *and S. warneri*, which were identified as *S. pseudintermedius*, *S. condimenti*, and *M. canis*, respectively, using molecular identification. The disagreement between *S. condimenti* and *M. canis* had low discrimination confidence levels in the VITEK^®^ reports. The discrimination confidence levels for 82 out of 104 isolates were excellent, 13 were very good, and seven isolates had good confidence levels. At species levels, the VITEK^®^ 2 system showed Kappa values of 0.972, 0.957, 0.795, and 1 for *S. pseudintermedius*, *S. schleiferi*, *S. aureus*, and *S. haemolyticus*, respectively. Positive and negative predictive values of the VITEK^®^ 2 system for identifying *S. pseudintermedius* in the studied population were 100% and 95.65%, respectively.

At the animal level (Table-2), based on the molecular methods, *S. pseudintermedius* was found in 50 dogs (89%), *S. schleiferi* in 10 dogs (18%), *S. aureus* in two dogs (4%), *S. haemolyticus* in four dogs (7%), and *S. condimenti* in one dog (2%). Table-2 lists the isolates found in infected dogs. Further, 45 dogs (80%), 10 dogs (18%), and one dog (2%) were simultaneously infected with one, two, and three isolates, respectively.

**Table-2:** Single and coinfections of Staphylococci, as identified by molecular method, in 56 canine pyoderma cases from September 2019 to September 2020 at Veterinary Teaching Hospital, Khon Kaen University.

Identified bacteria	Number of dogs (%)
Single infection	
*S. pseudintermedius*	40 (71.4)
*S. schleiferi*	3 (5.3)
*S. haemolyticus*	2 (3.6)
Coinfections	
*S. pseudintermedius* and *S. aureus*	2 (3.6)
*S. pseudintermedius* and *S. schleiferi*	7 (12.5)
*S. pseudintermedius* and *S. haemolyticus*	1 (1.8)
*S. haemolyticus, S. condimenti and* *M. canis*	1 (1.8)

*S. pseudintermedius=Staphylococcus pseudintermedius, S. schleiferi=Staphylococcus schleiferi, S. haemolyticus=Staphylococcus haemolyticus, S. aureus=Staphylococcus aureus, S. condimenti=Staphylococcus condiment, M. canis=Macrococcus canis*

### Identification of MRS using automated machine VITEK^®^ 2 system and *mecA* PCR

Results of the oxacillin susceptibility test using the VITEK^®^ 2 system showed that 36 MRS isolates included 24 MRSP, six methicillin-resistant *Staphylococcus schleiferi*, five methicillin-resistant *Staphylococcus haemolyticus*, and one methicillin-resistant *Staphylococcus* *warneri* isolate. Although *mec*A was found in all MRS isolates, only 51 of 68 MSS isolates contained *mec*A, based on the PCR results (Table-3).

**Table-3:** Detection of *mecA* among the phenotypically identified methicillin-resistant and methicillin-susceptible Staphylococci from canine pyoderma cases presented at VTH-KKU during September 2019 to September 2020.

Phenotypic identification	Methicillin-susceptible staphylococci		Methicillin-resistant staphylococci		Total
	
	*mecA*-positive	*mecA*-negative	*mecA*-positive	*mecA*-negative	
*S. pseudintermedius*	42	15	24	0	81
*S. schleiferi*	5	2	6	0	13
*S. aureus*	4	0	0	0	4
*S. haemolyticus*	0	0	5	0	5
*S. warneri*	0	0	1	0	1
Total	51	17	36	0	104

*S. pseudintermedius=Staphylococcus pseudintermedius, S. schleiferi=Staphylococcus schleiferi, S. aureus=Staphylococcus aureus, S. haemolyticus=Staphylococcus haemolyticus, S. warneri=Staphylococcus warneri,* VTH-KKU=Veterinary Teaching Hospital, Khon Kaen University

### Prevalence and risk factor of MRS in 56 dogs

Based on the phenotypic techniques, the MRS was found in 20 dogs. As shown in Table-4, univariate analysis using the Chi-square test showed that only one risk factor was significantly associated with MRS infection; treatment with antibiotics in the past 6 months with the odds ratio (OR) value at 3.714, 1.175–11.740 (95% confidence interval for OR).

**Table-4:** Univariate analysis of factors associated with methicillin-resistant Staphylococci infections among the 20 pyoderma dogs.

Factors	n	MRS (+) (%)	p-value
The dog finished the antibiotic as described			
Yes	38	16 (42.1)	0.147
No	18	4 (22.2)	
Total	56	20 (35.7)
Dog has pyoderma more than 1 time in the past 2 years			
None	11	2 (18.2)	0.146
1–2 times	14	6 (42.9)	
3–5 times	14	3 (21.4)	
More than 5 times	17	9 (52.9)	
Total	56	20 (35.7)
Allergies			
Yes	35	15 (42.9)	0.150
No	21	5 (23.8)	
Total	56	20 (35.7)
Dog received immune suppress drugs			
Yes	18	6 (33.3)	0.798
No	38	14 (36.8)	
Total	56	20 (35.7)
Dog received antibiotic in the past 6 months			
Yes	25	13 (52.0)	**0.022**
No	31	7 (22.6)	
Total	56	20 (35.7)
Dog received antibiotic in the past 12 months			
Yes	30	14 (46.7)	0.066
No	26	6 (23.1)	
Total	56	20 (35.7)	

MRS=Methicillin-resistant Staphylococci, VTH-KKU=Veterinary Teaching Hospital, Khon Kaen University. The significant p-value is given in bold form.

### Antibiotic susceptibility test and antibiogram of the *S. pseudintermedius* isolates

As 77.8% of the identified bacteria were *S. pseudintermedius*, their susceptibility to commonly used antibiotics for canine pyoderma was tested (Table-5) [16]. Of the tier 1 antibiotics, 2/57 MSSP isolates showed resistance to cephalothin and amoxicillin/clavulanic acid. A high proportion of MSSP (17/57) and MRSP (16/24) isolates were resistant to trimethoprim/SXT. Among the tier 2 antibiotics, at least 6/57 and 19/24 of MSSPs and MRSP isolates, respectively, were resistant to fluoroquinolone. An antibiogram of MRS isolates (Table-6) indicated that all MRSP and MRSW isolates were MDR. Furthermore, 2/6 MRSS and 2/5 MRSH isolates also had MDR characteristics.

**Table-5:** Results from Vitek^™^ antibiotic susceptibility test, as classified by Hillier *et al*.[16] of 81 *S. pseudintermedius* isolates from 56 canine pyoderma cases presented at VTH-KKU during September 2019 to September 2020.

Antibiotics	Methicillin-susceptible *S. pseudintermedius* (57 isolates)			Methicillin-resistant *S. pseudintermedius* (24 isolates)		
	
	Susceptible (%)	Intermediate (%)	Resistant (%)	Susceptible (%)	Intermediate (%)	Resistant (%)
Tier 1						
Cephalothin	55 (96.5)	0 (0)	2 (3.5)	0 (0)	0 (0)	24 (100)
Amoxicillin/Clavulanic acid	55 (96.5)	0 (0)	2 (3.5)	8 (33)	0 (0)	16 (67)
Trimethoprim/Sulfamethoxazole	40 (70.2)	0 (0)	17 (29.8)	8 (33)	0 (0)	16 (67)
Clindamycin	49 (86.0)	0 (0)	8 (14.0)	2 (8)	0 (0)	22 (92)
Tier 1 or 2						
Cefovecin	55 (96.5)	2 (3.5)	0 (0)	0 (0)	0 (0)	24 (100)
Cefpodoxime	55 (96.5)	1 (1.8)	1 (1.8)	0 (0)	0 (0)	24 (100)
Tier 2						
Doxycycline	31 (54.4)	0 (0)	26 (45.6)	0 (0)	0 (0)	24 (100)
Minocycline	32 (56.1)	3 (5.3)	22 (38.6)	0 (0)	2 (8)	22 (92)
Chloramphenicol	46 (80.7)	1 (1.8)	10 (17.5)	1 (4)	4 (17)	19 (79)
Erythromycin	45 (78.9)	0 (0)	12 (21.1)	1 (4)	0 (0)	23 (96)
Enrofloxacin	49 (86.0)	2 (3.5)	6 (10.5)	1 (4)	4 (17)	19 (79)
Marbofloxacin	50 (87.7)	0 (0)	7 (12.3)	1 (4)	0 (0)	23 (96)
Pradofloxacin	50 (87.7)	1 (1.8)	6 (10.5)	1 (4)	4 (17)	19 (79)
Gentamicin	50 (87.7)	6 (1.5)	1 (1.8)	2 (8)	11 (46)	11 (46)
Amikacin	55 (96.5)	0 (0)	2 (3.5)	24 (100)	0 (0)	0 (0)

*S. pseudintermedius=Staphylococcus pseudintermedius*, VTH-KKU=Veterinary Teaching Hospital, Khon Kaen University

**Table-6:** Antibiograms of 36 methicillin-resistant staphylococci isolated from 20 canine pyoderma cases presented at VTH-KKU during September 2019 to September 2020.

Classes of Antibiotics	Number of isolates	Beta-lactams						Macrolides	Fluoroquinolones		
		
		Oxacillin	Benzyl penicillin	Amoxicillin/clavulanic acid	Cephalothin	Cefpodoxime	Cefovecin	Erythromycin	Enrofloxacin	Marbofloxacin	Pradofloxacin
MRSP	2	R	R	R	R	R	R	R	R	R	R
MRSP	1	R	R	R	R	R	R	R	R	R	R
MRSP	2	R	R	R	R	R	R	R	R	R	R
MRSP	4	R	R	R	R	R	R	R	R	R	R
MRSP	2	R	R	R	R	R	R	R	R	R	R
MRSP	2	R	R	R	R	R	R	R	R	R	R
MRSP	2	R	R	R	R	R	R	R	R	R	R
MRSP	2	R	R	R	R	R	R	R	R	R	R
MRSP	2	R	R	R	R	R	R	R	R	R	R
MRSP	2	R	R	R	R	R	R	R	I	R	I
MRSP	2	R	R	R	R	R	R	R	I	R	I
MRSP	1	R	R	R	R	R	R	S	S	S	S
MRSS	2	R	R	R	NT	NT	R	S	R	R	I
MRSS	1	R	R	S	NT	NT	S	R	I	R	I
MRSS	1	R	R	S	NT	NT	S	R	I	R	S
MRSS	1	R	R	S	NT	NT	S	S	R	R	I
MRSS	1	R	R	S	NT	NT	S	S	I	R	I
MRSH	2	R	R	R	NT	NT	R	R	S	S	S
MRSH	1	R	R	R	NT	NT	R	R	S	S	S
MRSH	2	R	R	R	NT	NT	R	R	S	S	S
MRSW	1	R	R	R	NT	NT	R	R	R	R	R

**Classes of Antibiotics**	**Number of isolates**	**Lincosamides**	**Tetracyckines**		**Sulfonamides**	**Chloramphenicol**		**Aminoglycosides**		**Nitrofurans**	
					
		**Clindamycin**	**Doxycycline**	**Minocycline**	**Trimethoprim/Sulfamethoxazole**	**Chloramphenicol**	**Florphenicol**	**Gentamicin**	**Amikacin**	**Nitrofurantoin**	

MRSP	2	R	R	R	S	R	R	R	S	R	
MRSP	1	R	R	R	R	R	R	R	S	S	
MRSP	2	R	R	R	R	R	S	R	S	S	
MRSP	4	R	R	R	S	R	S	R	S	S	
MRSP	2	R	R	R	R	R	S	I	S	S	
MRSP	2	R	R	R	R	S	S	S	S	S	
MRSP	2	R	R	R	S	R	S	I	S	S	
MRSP	2	S	R	R	R	R	S	I	S	S	
MRSP	2	R	R	R	R	S	S	I	S	S	
MRSP	2	R	R	I	R	S	S	R	S	S	
MRSP	2	R	R	R	R	S	S	I	S	S	
MRSP	1	R	R	R	R	S	S	I	S	S	
MRSS	2	S	S	S	S	S	S	S	S	S	
MRSS	1	R	S	S	S	S	R	I	S	S	
MRSS	1	R	S	S	S	S	R	I	S	S	
MRSS	1	S	S	S	S	S	R	S	S	S	
MRSS	1	S	S	S	S	S	R	S	S	S	
MRSH	2	S	S	S	S	S	S	S	S	S	
MRSH	1	R	S	S	S	S	S	S	S	S	
MRSH	2	R	S	S	S	S	R	S	S	S	
MRSW	1	R	I	R	R	S	S	S	S	S	

R: Resistant, I: Intermediate, S: Susceptible, NT: Not tested

## Discussion

*Staphylococcus pseudintermedius* was the major bacteria found in dogs with pyoderma at the VTH-KKU, and 23% of all bacterial isolates were MRSP. The major risk factor associated with MRSP in the dogs was the recent use of antibiotics within the past 6 months. These bacteria are one among the three CoPS pathogens, along with *S. schleiferi* and *S. aureus*. It is commonly found in humans and animals and typically infects animal skin, provoking local or systemic infections [22]. However, *S. pseudintermedius* and *S. schleiferi* were more frequently associated with canine pyoderma than *S. aureus* [8]. Our study found a similar prevalence: more than 90% of isolates were *S. pseudintermedius* and *S. schleiferi*. Approximately 5% of the isolates were CoNS species. This is consistent with the prevalence of skin and soft-tissue infections of canine patients in a few other countries [14, 32, 33]. Automated methods have been increasingly used to characterize CoNS [33].

Methicillin-resistant *Staphylococcus pseudintermedius* was the most frequently identified pathogen in dogs, followed by MRSS and MRSA [8, 22], all of which belong to the CoPS group. Our study revealed similar results as the prevalence of MRSP was the highest, followed by MRSS. Several studies have also reported the presence of coagulase-negative MRS [18, 33, 34]. The two CoNS MRS isolated in this study were MRSH, which had the highest prevalence, and MRSW. Methicillin-resistant Staphylococci, especially MRSP, are usually resistant to β-lactam antibiotics and other antibiotic classes and exhibit MDR [8, 22]. This report aligns with the findings of our study; the isolated MRS also had MDR characteristics.

Our findings indicate that a very high proportion (81.6%) of MRS were MDR; all MRSP and 5/12 other MRS isolates. Moreover, MRS was found to be highly prevalent in VTH-KKU, especially MRSP. The identified risk factor for MRSP and other MRS in this study and by Loncaric *et al*. [18] was that the dog received antibiotics in the past 6 months. The association of other risk factors, such as an unfinished course of prescription, underlying diseases including allergies, or using prednisolone or other drugs affecting the antibiotic efficacy, with the population of MRSP-carrying dogs at the VTH-KKU during the study period, was small.

This study identified MRSS in 7% of patients which is lower than the prevalence in Bangkok [22]. Methicillin-resistant *Staphylococcus haemolyticus* was detected in four dogs (7%) and MRSW in one dog (2%) among the isolated CoNS identified by VITEK. Using multiple antibiotics frequently to treat Staphylococcal infections might increase the incidence of MRCoNS in dogs [35]. However, this study did not identify any MRSA, unlike a survey conducted in Bangkok that discovered 1% MRSA in the sampling dogs. Methicillin-resistant *S. aureus* is more prevalent in humans, possibly exposed to pet animals. Moreover, the MRSA isolated from dogs originated from humans [36]. The variation in owner populations between Bangkok and Khon Kaen might represent the presence of MRSA in their pets.

In the antibiogram, we observed that MRS isolates, especially MRSP, were resistant to other antibiotics; some were resistant to more than three classes of antibiotics. These isolates potentially acquire resistance via horizontal gene transfer. A previous report mentioned that *Staphylococcus* species served as reservoirs of AMR genes. Genetic exchange between isolates from different *Staphylococcus* species and their sources are associated with mobile genetic elements and biofilm formation [6].

In addition to β-lactams, 24/67 MRSP isolates were resistant to three or more classes of antibiotics and hence classified as MDR *S. pseudintermedius* (MDR-SP). In Bangkok-Thailand, MDR-SP was found in 59/64 MRSP dogs [22] and 28/28 MRSP dogs [37]. In this study, four MRSS isolates were MDR *S. schleiferi* (MDR-SS). The MRSW identified in our research were also MDR. Some studies have reported an increase in the prevalence of MRS CoNS in animals and humans [18, 33–35]. Although MRSA was not detected in this study, the proportion of MDR Staphylococci observed was alarmingly high.

Methicillin (oxacillin)-susceptible *mec*A-positive Staphylococci have been extensively identified worldwide [38]. Of the 87 isolates detected as methicillin-resistant using *mec*A-PCR, only 36 were found to be methicillin-resistant using the VITEK^®^ 2 system. The phenotypic expression of methicillin resistance could be related to the regulatory genes. The *mec*A and *bla*Z genes in Staphylococci facilitate resistance to β-lactam antibiotics. The *mec*A gene encodes PBP2a which has a weak affinity for β-lactams, whereas the *bla*Z gene encodes for β-lactamase [39]. These genes contain *mec* and *bla* regulatory genes called *mec*R1-*mec*I and *bla*R1-*bla*I, which encode inducer and repressor that synergistically control PBP2a production [40]. Liu *et al*. [41] revealed that the methicillin-susceptible phenotype in MRS isolates without *mec* regulators is due to the high expression of *bla*I, which can suppress *mec*A expression. Many MRS methicillin-susceptible isolates have been reported to comprise class B or class C *mec* complexes containing an intact *mec*A without *mec*R1 and *mec*I [42, 43]. The other factors related to the phenotypic expression of methicillin resistance are the mutations in c-di-AMP cyclase DacA, RelA, and Fem proteins [44–46].

The MDR-S antibiogram showed that some isolates were susceptible to amikacin, which has serious side effects, including nephrotoxicity, and requires close monitoring for kidney function [47]. Therefore, topical therapy is the only option for skin infection with MDR-SP, MRSP, and other MDR MRs [16, 48, 49]. Notably, among the tier 1 antibiotics for typical SP, the β-lactam antibiotics had a very high susceptibility, greater than but potentiated sulphonamides was at 70%. Therefore, prescribing β-lactams, such as amoxicillin-clavulanate, should be the first choice for treating canine pyoderma in these populations.

Due to its easy application, time- and cost-effectiveness, the VITEK^®^ 2 system is being increasingly used in everyday practice [50]. This system efficiently identifies Staphylococci species, MRS, and other bacterial species [33, 51–53]. In our study, the Kappa analysis showed almost perfect agreement between the VITEK^®^ 2 system and molecular methods for overall identification. The MIC result from VITEK helps select the most effective antimicrobial for the isolated pathogenic bacteria [54], identifies the methicillin-resistant and MDR bacteria, and is less time-consuming.

Our findings indicate that 81.6% of MRS were MDR, which included all MRSP and 5/12 MRS isolates. Although MRSA was not found in this study, MDR Staphylococci were highly prevalent. Methicillin-resistant *S. pseudintermedius* showed high resistance to tier-1 antibiotics, including SXT and clindamycin, and most tier-2, including fluoroquinolones. The only antibiotic to which these bacteria were susceptible was amikacin. These results provide information to the wider community, including the government, veterinarians, and various related parties. They can serve as basic information for further research or national policymaking related to antimicrobial stewardship to prevent or reduce the emergence of new cases of MRS infection in Thailand.

## Conclusion

A high prevalence of MRS and MDR was observed in the referral center. The likelihood of this occurrence is that owners did not follow the instructions of giving antibiotics properly, as well as the possibility of horizontal gene transfer existence. The use of antibiotics at the primary care unit should be discussed, both for veterinarians and pet owners, to prevent the occurrence of AMR in Thailand.

## Authors’ Contributions

PASP: Laboratory work, data analysis, interpretation of data, and original draft preparation. PP and SJ: Methodology, supervision, validation, and editing manuscript. JK: Conception of the study, methodology, clinical sampling, supervision, and editing of the manuscript. All authors have read, reviewed, and approved the final manuscript.
